# The Procalcitonin-guided Antibiotics in Respiratory Infections (PARI) project in general practice – a study protocol

**DOI:** 10.1186/s12875-022-01646-6

**Published:** 2022-03-12

**Authors:** Nadia Filipsen, Holger Bro, Lars Bjerrum, Jens-Ulrik Staehr Jensen, Rune Aabenhus

**Affiliations:** 1 Department of Public Health, Section of General Practice, University of Copenhagen, Copenhagen, Denmark; 2 Research Unit of General Practice, University of Copenhagen, Copenhagen, Denmark; 3 General Practice, Naestved Laegecenter, Naestved, Denmark; 4 Department of Internal Medicine, Herlev and Gentofte University Hospital, Copenhagen, Denmark

**Keywords:** Procalcitonin, Point-of-care test, Antibiotic use, General practice, Acute respiratory tract infection

## Abstract

**Background:**

Antibiotic resistance is a global health challenge and the close correlation between antibiotic use and the development of resistance makes it essential to maintain a rational use of antibiotics. Most antibiotics are prescribed in general practice against acute respiratory tract infections (ARTI), even though most of these infections are of viral etiology. Thus, a safe method to substantially reduce unnecessary use of antibiotics in general practice is needed. Procalcitonin (PCT) is a precursor protein with very low circulating levels in the blood under physiological conditions. However, in response serious bacterial infection the level of PCT in the blood may increase significantly. Until recently, quantitative analyses of PCT was performed in hospital laboratories, impeding the implementation of PCT in primary care. Our aim is to determine whether it is possible to lower the use of antibiotics in patients presenting with symptoms of ARTI, without significantly prolonging the period of illness, by using a newly released PCT point-of-care test in general practice.

**Methods:**

The Procalcitonin-Guided Antibiotics in Respiratory Infections (PARI) study is a randomized, single-blinded, non-inferiority, multi-practice intervention study comparing a PCT-group to a control group. Patients (*N* = 508) will be randomly assigned 1:1 to standard care or to the PCT group. The primary outcomes the duration of illness and symptoms from ARTI measured with the Acute Respiratory Tract Infection Questionnaire. Secondary outcomes include (1) Number of participants in each trial arm exposed to antibiotic treatment at index consultation (day 1) and within 30 days, (2) Number of participants in each trial arm with side effects from antibiotic treatment within 14 days, (3) Number of participants in each trial arm with re-consultations within 30 days, (4) Number of participants in each trial arm admitted to hospital (including diagnosis and mortality) within 30 days, (5) Characterization of biomarker (CRP and PCT) level at index consultation. Tertiary outcomes include patient and general practitioner satisfaction with the use of the PCT point-of-care test, and long-term follow-up.

**Discussion:**

To our knowledge, this is the first study to examine a PCT point-of-care test in general practice with the aim of reducing the use of antibiotics in patients with symptoms of ARTI. Results of this study may prove important in targeting antibiotic treatment only to those patients who need it, thus contributing to limiting the spread of antibiotic resistance.

**Trial registration:**

ClinicalTrials.gov Identifier: NCT04216277, date of registration: 2. of January 2020.

## Background

According to the World Health Organization (WHO), antibiotic resistance is a major threat to global health, food security and development [1] which is a serious concern as effective antibiotics are one of modern medicine’s most necessary and lifesaving treatments.

There is close correlation between the use of antibiotics and the development of antibiotic resistance. The primary care sector (not hospital setting) is responsible for most antibiotic prescriptions (approximately 90%) in Denmark, with general practice being responsible for three quarters of these [2]. Therefore, a rational use of antibiotics in general practice is essential in preventing antibiotic resistance. Furthermore, the benefits from rational use of antibiotics also include prevention of possible drug toxicities and conditions such as C. difficile-mediated diarrhea.

One of the main indications for antibiotic use in general practice are acute respiratory tract infections (ARTI) [3, 4]. However, most ARTIs are of viral etiology and antibiotics are often not needed for the patient to recover [5, 6] and may have many negative impacts to both the patient and to society [7, 8]. In selected cases macrolide antibiotics are prescribed by GPs because of their possible anti-inflammatory properties which may be independently beneficial, aside from their antibacterial action [9].

There are at least two major considerations when assessing the appropriateness of antibiotic prescribing in general practice: professional clinical judgement and patient expectations. The general practitioner (GP) must assess several findings such as symptoms, objective findings and paraclinical examinations including C-reactive protein (CRP) levels, leucocyte count or chest x-ray. None of these are specific to bacterial infection [10, 11] and clinical uncertainty regarding management may lead to unjustified prescribing. Pressure from patients may also prompt antibiotic prescriptions [12]. These two considerations may interact.

Most Scandinavian GPs are familiar with the use of CRP measurement and it has been shown that use of CRP guidance can decrease the use of antibiotics for ARTI in general practice [13]. However, CRP is not specific to bacterial infections and CRP levels also rise in cases of other etiologies such as surgery, rheumatic inflammation, viral diseases, systemic illnesses and cancer [14].

Procalcitonin (PCT) is a precursor protein that occurs at very low levels in the blood under physiological conditions [15]. It is, however, correlated with high levels in the blood in cases of serious bacterial infection. It also has a shorter time of release, a shorter half-life than CRP and it is less sensitive to surgery and non-bacterial infection [16, 17]. Studies from general practice have to date shown a reduction in the prescription of antibiotics by PCT-guided antibiotic treatment without significantly prolonging the duration of restrictions in patients’ daily lives due to infection [18, 19]. However, these studies were performed without a PCT point-of-care(POC) test and the test result was not available until hours after the initial patient assessment because it was sent for analysis in a hospital laboratory. Further, these studies were performed in an era when CRP-guided antibiotics were not systematically established.

To our knowledge, the Procalcitonin-guided Antibiotics in Respiratory Infections (PARI) trial is the first to examine a PCT POC test in general practice. The POC element of the intervention may be of crucial importance, since it makes a PCT-guided antibiotic intervention logistically realistic in a primary care setting for the first time.

The aim of this study is to determine whether it is possible to lower the use of antibiotics in patients presenting to their GP with symptoms of ARTI, without significantly prolonging the period of illness by using a PCT POC test.

The PARI study aim is in line with Danish national goals to improve the rational use of antibiotics in general practice [20].

## Methods/Design

### Design, setting and randomization

This study is a randomized, single-blinded, non-inferiority, multi-practice intervention study comparing a PCT group to a control group. We expect the mean duration of ARTI symptoms to be approximately 9 days [21, 22] and we want to detect if this increases to > 10 days in the PCT group i.e. a non-inferiority limit of one day.

The PARI project is a single-blinded study as participants will not be informed of their allocation group: standard care or PCT group. GPs will have access to the PCT results for the intervention group. GPs can tell patients if the biomarker suggests whether antibiotics are needed or not. This is based on the CRP results for both groups and, in the intervention group only, also the PCT levels. Patients will be informed only that “the biomarker” suggests relevant treatment, and other similar generic statements; they will not be informed as to which biomarker the physician refers.

In case of accidental demasking, the investigator will report this as a protocol deviation.

If the PCT level is above 2 ng/L, demasking will deliberately take place because the GP will have to consider referring the patient to hospital on suspicion of serious infection.

At least five practices in two different regions of Denmark, The Capital Region and Region Zealand, will recruit participants, representing both urban and rural areas.

Randomization will be performed by a remote internet-based randomization tool (sealedenvelope.com). The allocation sequence will be stratified by site, age and sex.

### Participants and procedures

**Fig. 1:**
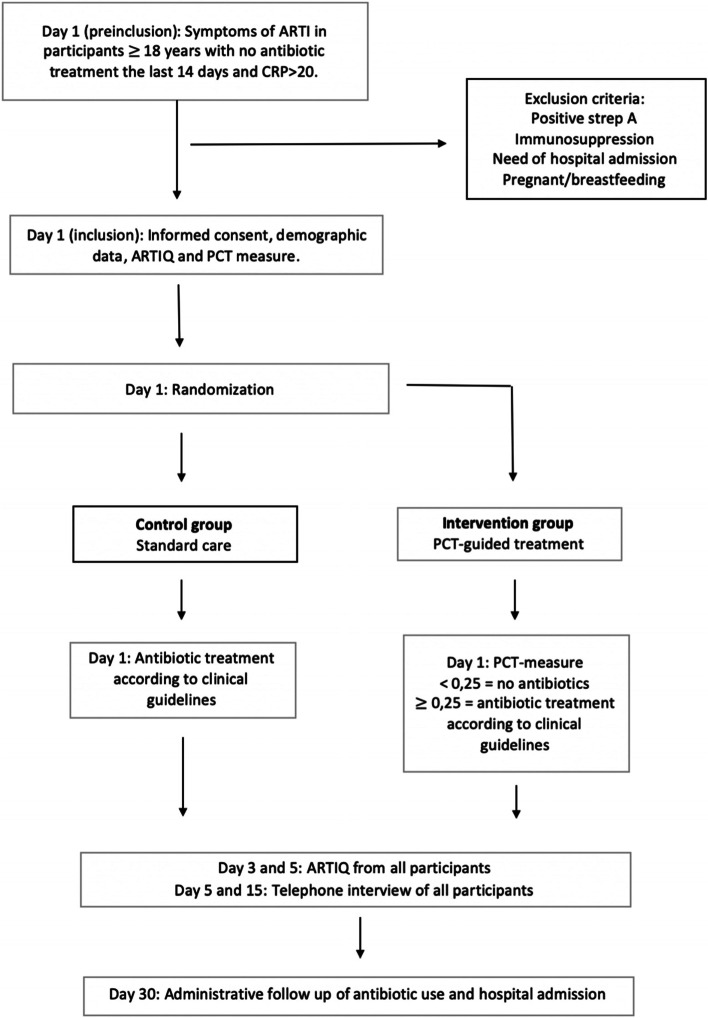
Flow chart of the PARI study

Figure 1; Table 1 present a flow chart detailing the enrollment, interventions and assessments.

**Table 1:** Table 1 Shows the interventions in both the intervention and the control group at days 1, 3, 5, 7 and 15. They are further explained below

Evaluation	Day				
	**1**	**3**	**5**	**7**	**15**
**Inclusion, CRF, PCT measurement**	**x**				
**ARTIQ questionnaire**	**x**	**x**	**x**	**x**	**x**
**Telephone interview**			**x**		**x**

*CRF* Case Report Form, *ARTIQ* Acute Respiratory Tract Infection Questionnaire

Adult patients presenting to their GP with symptoms of ARTI will be recruited.

#### Inclusion criteria

The following inclusion criteria must be present:

1. Age $$\ge$$ 18 years
2. Ability to read, understand and willingness to give written consent to participate in the study.
3. Symptoms of acute upper/lower respiratory tract infection (pharyngitis, tonsillitis, otitis media, exacerbations of chronic obstructive pulmonary disease (COPD), or asthma and pneumonia).
4. CRP > 20 mg/mL (based on POC-test in clinic)..

#### Exclusion criteria

If one or several of the following criteria are present, the participant will be excluded from the study:

1. The patient is being followed up by a hospital because of verified immune defect or the patient is currently neutropenic (neutrophils < 0,5 mia/L during the last 7 days).
2. The patient has a positive strep A test, (only performed if indicated according to Centor criteria [23].
3. The patient has been treated with antibiotics during the last 14 days up to inclusion.
4. The doctor estimates that the patient needs to be admitted to hospital.
5. The patient is pregnant or breastfeeding..

**Fig. 2:**
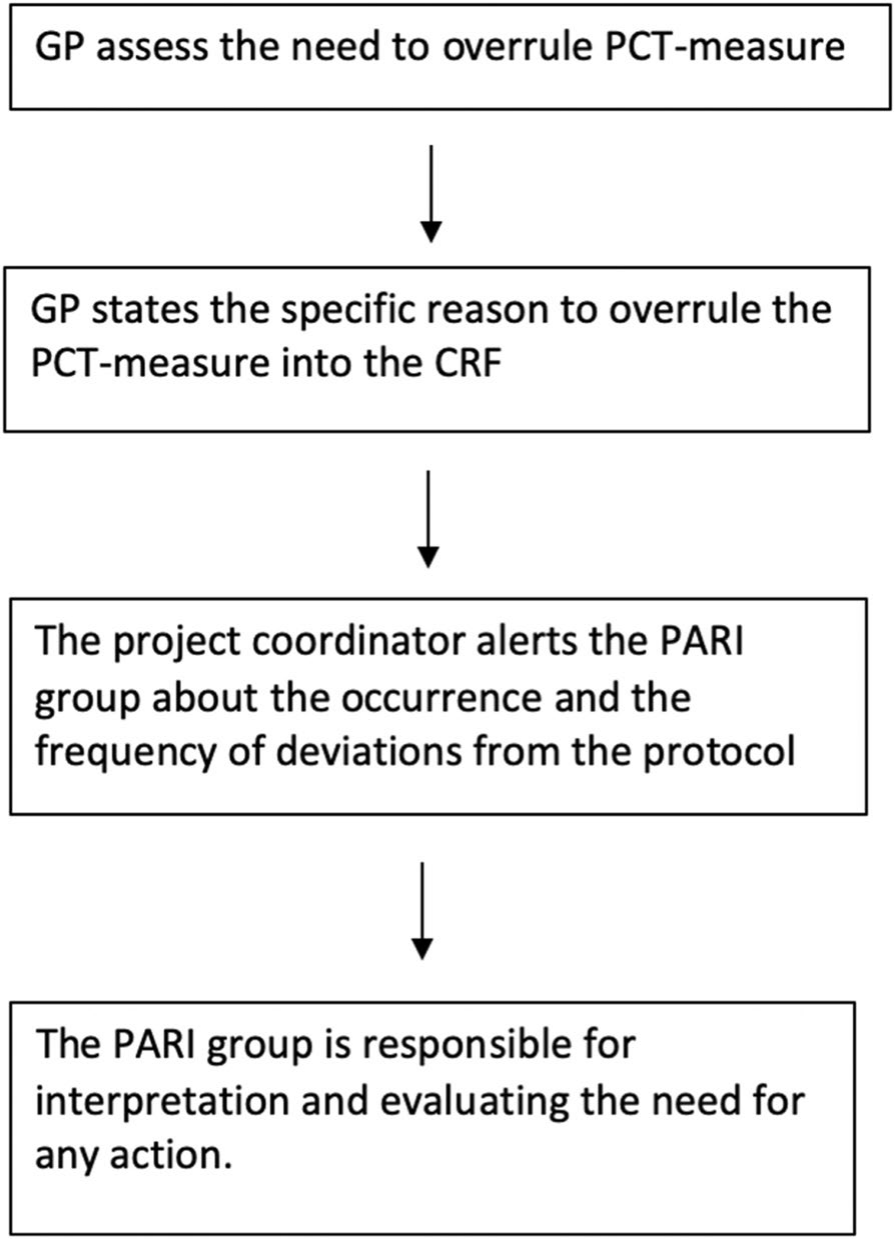
Algorithm in case of deviation from the protocol intervention

At any point, the GP will be able to overrule the PCT measurement in the intervention group based on clinical judgement. The reason for deviating from the suggested algorithm must be stated (see Fig. 2).

#### Interventions and timeline

All participants will receive standard care as per the GPs’ normal procedures and treatments including POCT CRP testing. Patients randomized to the intervention/PCT group will additionally receive diagnostic testing with measurement of PCT.

#### Day 1

Following pre-inclusion assessment, including oral and written information about the PARI study, eligible patients must sign a consent form prior to participation. Subsequently, they will be randomized to either (i) the control group, or (ii) the PCT group. Data-gathering on day 1 will cover participant demographics, the current infection and the likely focus, duration of illness, and assumed etiological micro-organism, objective findings and CRP-level, and results of a self-reported acute respiratory tract infection questionnaire (ARTIQ). In addition, PCT values for the intervention group will be collected on day 1. The control group will receive standard care according to clinical guidelines [24, 25], and the intervention group will receive standard care *plus* PCT-guided treatment (see below).

#### Days 3 and 7

Each participant will be sent a link to the ARTIQ by e-mail on days 3 and 7 (it will also be available in hard copy if required, with responses collated by the investigator). The participants enter their responses into a database (Surveyxact). The content of the ARTIQ is presented in Appendix 1.

#### Days 5 and 15

All participants will be interviewed over the telephone to follow-up on their clinical condition (changes in general condition, fever, pain or coughing), days unable to work, the results of the ARTIQ, and compliance.

#### Day 30

An administrative follow-up to gather information about the use of antibiotics and hospital admissions.

### Outcomes

#### Primary outcome

Duration of illness and symptoms from an ARTI. The participant-reported primary outcome will be assessed as the number of days until a patient’s daily activities (e.g. work and recreation) are no longer restricted by symptoms from an ARTI. The non-inferiority margin between the intervention group and the control group is set at a one-day difference. The recovery measure will be the specific day indicated by the participants using the validated ARTIQ.

#### Secondary outcomes

These are the outcomes to be assessed regarding antibiotic treatment and clinical measures of treatment failure:

1. Number of participants in each trial arm exposed to antibiotic treatment at index consultation (day 1) and within 30 days.
2. Number of participants in each trial arm with side effects from antibiotic treatment within 14 days.
3. Number of participants in each trial arm with re-consultations within 30 days.
4. Number of participants in each trial arm admitted to hospital (including diagnosis and mortality) within 30 days.
5. Characterization of biomarker (CRP and PCT) level at index consultation..

#### Tertiary outcomes

These outcomes relate to patient and GP satisfaction with the treatment, the use of the PCT POC test, and the long-term follow-up. This will be measured in semi-qualitative studies.

### Analysis of procalcitonin

The PCT POC test (ThermoFisher Scientific® PCT Direct, Berlin, Germany) has a functional assay sensitivity (FAS) of 0,22 ng/mL, which is within target range of application in general practice.

The test has two parts: (1) a measuring device (immunochromatography) which analyzes the PCT level in 20 µl of blood (capillary); and (2) a device with a needle to take out 50 µl of blood from the finger and that can be placed directly into the measuring device. Analysis takes 20 min and the only discomfort for the patient is from taking the capillary blood sample from the finger.

#### Guidance for PCT levels

PCT levels are guided by current literature [16, 17, 26] as follows:

- PCT-levels between < 0,25 ng/mL: low risk of bacterial etiology, antibiotic treatment will not be prescribed.
- PCT levels $\ge$ 0,25: bacterial etiology is more likely, antibiotic treatment will be prescribed

### Statistical methods

#### Data management

Data from the study in the form of Case Report Forms (CRFs), questionnaires and telephone interviews will be collected in Surveyxact and transferred to SAS (v. 9.4. Cary, NC, US) for analyses. Raw data will be anonymized in the practices before being imported into the data matrices by replacing names and civil registration numbers with study numbers as part of the randomization process.

Collection, storage and processing of data has been approved by Data Protection Systems (in Danish: “Datatilsynet”).

#### Sample size

A comparable study by Briel et al. [18] showed that the number of days when patients experienced restrictions from an ARTI had a standard deviation of 4 days. The PARI project’s non-inferiority design allows for a clinical non-important difference in sickness of max. 1 day in the PCT group compared to the standard care group. We expect an ARTI to last on average 9 days [21, 22]. The power (1-β) is set at 85% and α at 0.025 (non-inferiority study design). We believe these are relevant estimates since non-inferiority of a PCT-guided antibiotic treatment has previously been demonstrated [18, 19].

The two arms of the study will be randomized 1:1 with 231 participants in each arm. We estimate that 10% of participants will not complete the project, so a total of 508 participants will be needed.

#### Statistical analysis

An intention-to-treat (ITT) and a per-protocol (PP) analysis are planned. Chi-square tests for binary effects and t-test or Mann-Whitney U-test for interval scale effects will be used (depending on distribution).

We will also present descriptive statistics in the different subgroups such as sex, age, and demographic variables.

#### Safety

If the participant’s condition gets worse they will contact their own doctor or the doctor on call as in every other incidence.

Serious unexpected events and unexpected events that that occur during the study, regardless of allocation group, will be reported orally and in writing to the project coordinator who is responsible for follow-up on unexpected events.

The safety issues will be discussed at all PARI study board meetings.

### Ethical issues

The study and all participant-related information have been approved by the regional ethics committee.

All of the interventions being performed in the PARI study are already known and tested in previous trials or approved treatments.

The measurement of PCT level is done by way of a finger prick test collecting a small blood sample of approximately 20 µl. This kind of blood sampling is a common procedure in general practice, and troublesome unexpected events as a direct result of this procedure are unlikely to happen.

Unexpected events as a result of potential overtreatment with antibiotics are also not considered likely to happen in the context of already published literature [18, 19, 27].

### Time plan

The PARI project recruited the first patient in February 2020. It was estimated that recruitment of all patients would take 18 months. However, the COVID-19 pandemic has paused the study. In Denmark, patients with symptoms of ARTI were triaged by phone or sent directly to COVID-19 clinics in hospitals. Therefore, it is still unclear when we will be able to continue recruiting participants to the PARI project, however, it is expected fall/winter 2021.

## Discussion

The PARI project is, to our knowledge, the first study to examine a PCT POC test in general practice. Previous studies have examined PCT levels either in a hospital setting or in general practice with a venous blood sample and a prolonged wait for the result. GPs are familiar with POC tests (such as CRP and strep A tests) and these are valuable tools for decision-making in many cases. However, application of PCT as an add-on when an ARTI is not trivial (e.g. CRP values are above 20 mg/L), may aid the GP in ruling out a serious bacterial infection. This could in theory limit GP uncertainties and assist in targeting antibiotics only to the relatively few patients with a high probability of having a bacterial infection and likely to benefit from antibiotic treatment. If this study shows that the PCT-guided antibiotic treatment for ARTI can reduce the use of antibiotics with no safety concerns for the patients (i.e. prove non-inferiority), it will contribute to reaching national goals for improving the rational use of antibiotics in general practice and thereby reduce the development of antibiotic resistance.

Strengths of the study include the randomized controlled trial design that will provide a clear answer regarding the study’s primary objectives. A non-inferiority design has been chosen to examine whether a PCT POC test is not worse than standard care regarding patient safety and duration of symptoms. In addition, we expect PCT testing to reduce the number of antibiotic prescriptions.

There are some limitations to the study. First, the study is single-blinded, i.e. patients are unaware of the treatment group to which they have been randomized. This is important to minimize reporting bias in the PARI trial as the primary outcome is a patient-reported outcome (recovery). It is impossible in this setup to blind GPs since they need to know which group the patient is randomized to as part of the clinical decision-making process. This information bias could lead to the GP learning from the results of the PCT test and thereby improving their clinical judgement. However, we do not believe such a bias will significantly change the results of the study and the direction of the bias would likely be conservative. Second, the GPs who opt to be included in this study may be more motivated and interested in research and they may be more reluctant to prescribe antibiotics in general, compared to other GPs. This kind of selection bias could affect the results. However, the direction of this bias is also likely to be conservative.

Another limitation to consider is that the analysis of a PCT POC test takes 20 min compared to the CRP measurement, which only takes a couple of minutes. In this study setting we do not consider this a problem, but in the longer term, it could affect the practical use and possible implementation of findings on a wider scale in general practice.

In conclusion, the PARI study is, to our knowledge, the first to examine a PCT POC test in general practice aimed at reducing the use of antibiotics in patients with symptoms of ARTI, without prolonging the symptoms and the duration of restrictions to patients’ daily lives. Since antibiotic resistance is a worldwide health problem, we believe that the results of this study could help to better target antibiotic treatment only to those patients with bacterial infections, which in turn will contribute to limiting antibiotic resistance.

## Data Availability

The PARI study steering committee agrees on data sharing and will accept requests for data sharing once the study is completed. The PARI steering committee will evaluate the scientific soundness of each proposed project and will grant access whenever projects seem scientifically sound. All data sharing will apply to national and international legislation, rules and other regulations by regional or national authorities. Applications for data require a formal application and will be decided upon the board of the PARI group.

## Abbreviations

POC test: Point-of-care test
PCT: Procalcitonin
GP: General practitioner
ARTI: Acute respiratory tract infection
ARTIQ: Acute respiratory tract infection questionnaire
CRP test: C-reactive protein test
PARI: Procalcitonin-guided antibiotics for respiratory infections
